# Protein Kinase B Controls Transcriptional Programs that Direct Cytotoxic T Cell Fate but Is Dispensable for T Cell Metabolism

**DOI:** 10.1016/j.immuni.2011.01.012

**Published:** 2011-02-25

**Authors:** Andrew N. Macintyre, David Finlay, Gavin Preston, Linda V. Sinclair, Caryll M. Waugh, Peter Tamas, Carmen Feijoo, Klaus Okkenhaug, Doreen A. Cantrell

**Affiliations:** 1 College of Life Sciences, Division of Cell Biology & Immunology, University of Dundee, Scotland DD1 5EH, UK; 2 Babraham Research Institute, Babraham Research Campus, Cambridge CB22 3AT, UK

## Abstract

In cytotoxic T cells (CTL), Akt, also known as protein kinase B, is activated by the T cell antigen receptor (TCR) and the cytokine interleukin 2 (IL-2). Akt can control cell metabolism in many cell types but whether this role is important for CTL function has not been determined. Here we have shown that Akt does not mediate IL-2- or TCR-induced cell metabolic responses; rather, this role is assumed by other Akt-related kinases. There is, however, a nonredundant role for sustained and strong activation of Akt in CTL to coordinate the TCR- and IL-2-induced transcriptional programs that control expression of key cytolytic effector molecules, adhesion molecules, and cytokine and chemokine receptors that distinguish effector versus memory and naive T cells. Akt is thus dispensable for metabolism, but the strength and duration of Akt activity dictates the CTL transcriptional program and determines CTL fate.

## Highlights

► Akt is dispensable for T cell metabolism ► Akt controls perforin and interferon gamma expression in CTL ► Akt controls CTL migration ► Akt controls cytokine receptor expression in CTL

## Introduction

Cytotoxic T cells that express CD8 and recognize peptide-class I major histocompatibility complexes play a key role in immunity. The transcriptional program that determines the effector or memory fate of CTL is controlled by the TCR and cytokines. In particular, interleukin-2 (IL-2), a member of the common cytokine receptor gamma-chain (γ_c_) family of cytokines, has a key role to maintain antigen-specific effector and memory CD8^+^ T cells during viral infections (Bachmann et al., 2007; D'Souza and Lefrançois, 2003; Kalia et al., 2010; Malek and Castro, 2010; Williams et al., 2006). IL-2 is important for CTL because it can upregulate glucose and nutrient uptake in a response that increases cellular energy production in order to support the biosynthetic demands of rapid cell division and effector function (Cornish et al., 2006; Maciver et al., 2008). However, IL-2 also directs the transcriptional program of CTL and promotes effector CTL differentiation at the expense of memory cell formation (Kalia et al., 2010; Obar et al., 2010; Pipkin et al., 2010).

IL-2 acts via a receptor complex consisting of the common gamma chain (γ_c_), a β subunit (CD122), and CD25. Triggering of the IL-2 receptor stimulates the Janus family kinases Jak1 and Jak3 and induces the tyrosine phosphorylation and DNA binding of STAT (signal transducer and activator of transcription) family transcription factors STAT5 and STAT3. The activation of STAT5 is important for IL-2 signaling (Malek and Castro, 2010) but is not sufficient to mimic the effects of IL-2 on T cell biology. In this context, IL-2 induces sustained accumulation of phosphatidylinositol 3,4,5-trisphosphate (PI(3,4,5)P_3_), the product of phosphatidylinositol 3-kinases (PI3K) (Cornish et al., 2006; Sinclair et al., 2008), and activates a cascade of serine-threonine kinases. These include Akt (protein kinase B [PKB]) (Waugh et al., 2009) and other members of the protein kinase A/G/C-related (AGC kinase) family including p70 ribosomal S6 kinases (S6Ks) and p90 ribosomal S6 kinase (RSK) (Lin et al., 2008). The proper balance of Akt and STAT5 signaling seems to be critical for virus-specific CD8^+^ T cells (Hand et al., 2010). However, the precise role of Akt in the transcriptional programs that direct effector CD8^+^ T cell differentiation has not been explored.

The coordination of glucose metabolism is essential for T cell activation (Cham et al., 2008; Maciver et al., 2008) and this function might be controlled by PI3K-Akt. This assumption stems partly from extrapolating data from thymus studies, where it is clear that PI3K and Akt control glucose uptake and the expression of amino acid transporters and transferrin receptors in T cell progenitors (Juntilla et al., 2007; Kelly et al., 2007; Mao et al., 2007). Further support for a tropic role for PI3K-Akt in peripheral T cells come from experiments with PI3K inhibitors, which prevent Akt activation and prevent increases in both glucose uptake and amino acid uptake by activated peripheral T cells (Cornish et al., 2006). However, there are caveats because some of the PI3K inhibitors used in early T lymphocyte studies (Cornish et al., 2006) have off-target effects (Bain et al., 2007). In this context, evidence for Akt-independent pathways that control T cell metabolism and proliferation are emerging (Bayascas, 2008; Finlay et al., 2009; Jacobs et al., 2008). Moreover, constitutively active Akt can stimulate promote cell growth and survival of CD4^+^ T cells but not CD8^+^ T cells (Hand et al., 2010; Rathmell et al., 2003; Saibil et al., 2007), indicating that Akt may not have an obligate role to control metabolism in all T cell subpopulations. Accordingly, the focus of the present study is the role of PI3K-Akt in TCR and IL-2 regulation of CTL biology. The data have established that PI3K- and Akt-independent mechanisms control CTL metabolism, survival, and proliferation but there is a seminal role for Akt signaling in CTL to direct a diverse transcriptional program that determines effector versus memory T cell fate.

## Results

### p110δ Couples the IL-2 Receptor to Akt but IL-2-Mediated T Cell Proliferation Is p110δ Independent

**Figure 1:**
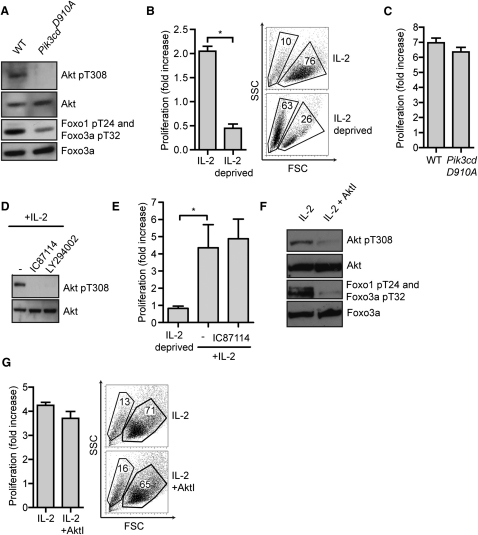
Akt Is Not Necessary for IL-2-Driven Survival or Proliferation in CTL (A) Immunoblot analysis of Akt T308 and Foxo1 T24, Foxo3a T32 phosphorylation in wild-type (C57/BL6) and p110δ^D910A^-expressing (*Pik3cd*^D910A^) splenic T cells activated with 2C11 for 48 hr then cultured in IL-2 for 3 days. (B) Wild-type splenic T cells were activated with 2C11 for 48 hr then cultured in IL-2 for 5 days, with some cells being deprived of IL-2 for the last 24 hr of culture. Data show fold change in cell number over the last 24 hr of culture and the cell FSC and SSC profiles at the end of the experiment. Gates indicate dead (left) and viable (right) cell populations. (C) Splenic T cells from wild-type and p110δ^D910A^-expressing mice (*Pik3cd*^D910A^) were cultured as in (A). Data show fold change in cell number over the last 48 hr of culture. (D) Western blot analysis of Akt T308 phosphorylation in P14 T cells activated with gp33 for 48 hr then cultured in IL-2 for 5 days, with IC87114 or LY294002 being added for the last 48 hr of culture. (E) P14 T cells were activated with gp33 for 48 hr then cultured in IL-2 for 5 days, with IC87114 added for the last 48 hr of culture. Alternatively, IL-2 was removed from the culture for the last 48 hr. Data show fold change in cell number over last 48 hr of culture. (F and G) Wild-type splenic T cells activated with 2C11 for 48 hr cultured in IL-2 for 5 days, with AktI being added for the last 48 hr of culture. Data show (F) immunoblot of Akt T308 and Foxo1 T24, Foxo3a T32 phosphorylations and (G) FSC and SSC profiles of cells at the end of the culture plus fold change in cell number during the last 48 hr of culture. Data are representative of 3 (A, C–F), 7 (B), or 11 (G) experiments; mean ± SEM of biological replicates.

TCR-primed CD8^+^ T cells cultured in IL-2 clonally expand and differentiate to effector cytotoxic T cells (CTL) (Manjunath et al., 2001). This model mimics the in vivo situation where sustained IL-2 signaling promotes the production of terminally differentiated effector cytotoxic T cells (Kalia et al., 2010; Malek and Castro, 2010; Pipkin et al., 2010). IL-2 strongly activates Akt and antigen-experienced T cells cultured in IL-2 contain high amounts of Akt phosphorylated on the 3-phosphoinositide-dependent kinase 1 (PDK1) substrate site T308 (Figure 1A). The TCR activates Akt via a PI3K complex that contains the p110δ catalytic subunit (Garçon et al., 2008). Figure 1A shows that p110δ PI3K also coupled the IL-2 receptor to Akt. Hence, T cells with wild-type p110δ substituted with a catalytically inactive mutant, p110δ^D910A^, do not phosphorylate Akt T308 in response to IL-2 and thus lack Akt activity as judged by the loss in phosphorylation of Foxo transcription factors (Figure 1A).

The sustained proliferation and survival of antigen-primed CTL is dependent on exogenous IL-2 (Figure 1B). However, activated p110δ^D910A^-expressing T cells proliferated normally to IL-2 (Figure 1C). Further experiments examined the impact of a p110δ inhibitor, IC87114, on IL-2-induced Akt activity in antigen-activated P14 TCR transgenic T cells. Triggering of the TCR expressed on these cells with a peptide from the lymphocytic choriomeningitis virus (LCMV), glycoprotein gp33-41, presented by the MHC class I molecule H-2D^b^, followed by clonal expansion in IL-2, generated a homogenous population of CTL. The exposure of IL-2-maintained P14 CTL to IC87114 caused loss of Akt T308 phosphorylation (Figure 1D) but did not prevent T cell proliferation (Figure 1E).

To explore further the role of Akt in IL-2 proliferative responses, a set of experiments with AktI, a selective allosteric inhibitor of all three isoforms of Akt, were performed. AktI prevents the essential PDK1-mediated phosphorylation of Akt on T308 (Green et al., 2008). The addition of AktI to CTL caused loss of Akt T308 phosphorylation and a resultant loss of Akt activity as judged by the loss in phosphorylation of the Akt substrates Foxo1 and Foxo3a (Figure 1F). However, AktI did not prevent IL-2-induced proliferation of the CTL nor did it cause cell death (Figure 1G). Hence, CTL do not need active Akt to proliferate and survive.

### PDK1 Is Required for T Cell Proliferation

**Figure 2:**
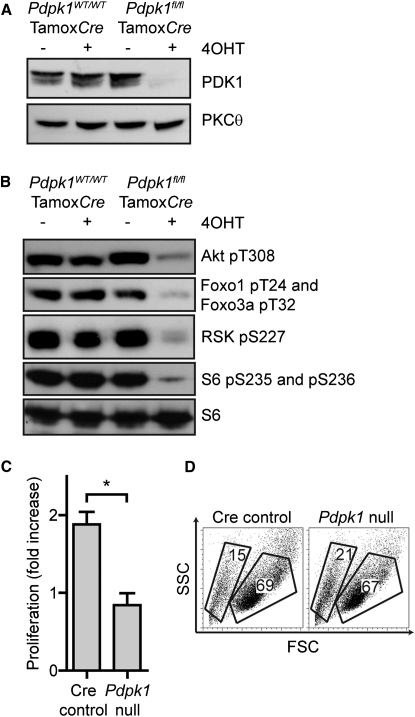
PDK1 Maintains IL-2-Driven Proliferation (A and B) Immunoblot analysis of (A) PDK1 expression and (B) phosphorylation of Akt, Foxo1 and Foxo3a, RSK, and small ribosomal subunit S6 in *Pdpk11^fl/fl /tm9(creEsr1)Arte^* (*Pdpk1^fl/fl^* Tamox*Cre*) and *Pdpk1^+/+/ tm9(creEsr1)Arte^* (*Pdpk1^WT/WT^* Tamox*Cre*) T cells activated with 2C11 for 2 days and then cultured in IL-2 for 5 days, supplemented as indicated with 4OHT for the final 72 hr of culture. (C and D) Fold change in cell number over the last 48 hr of culture (C) and FSC and SSC profiles (D) of 4OHT-treated cells from (A), gates indicate dead (left) and viable (right) cell populations. Data are representative of three (A, B) or five (C, D) experiments; mean ± SEM of biological replicates.

One explanation for the Akt independence of IL-2-induced proliferation is that other members of the AGC family of kinases might compensate for loss of Akt function. We therefore examined the impact of deleting the gene encoding PDK1, *Pdpk1,* on IL-2 signal transduction. PDK1 phosphorylates and activates multiple AGC kinases such as RSK and S6K1 in a response that is independent of PI(3,4,5)P_3_ production (Bayascas, 2008). *Pdpk1* deletion is thus a strategy that simultaneously inactivates Akt- and PI(3,4,5)P_3_-independent AGC kinases in T cells (Kelly et al., 2007). Accordingly, mice expressing floxed *Pdpk1* alleles (*Pdpk1*^fl/fl^) were backcrossed with C57BL/6-GT(ROSA)26^tm9 (creEsr1) Arte^ mice (TamoxCre) that express a tamoxifen-regulated Cre recombinase in the ROSA locus. *Pdpk1*^f*l/fl*^ Tamox*Cre* CTL were then generated and treated with 4-hydroxytamoxifen (4OHT) to delete the floxed *Pdpk1* alleles. *Pdpk1* deletion in 4OHT-treated *Pdpk1^fl/fl^* Tamox*Cre* CTL was confirmed (Figure 2A). Importantly, immunoblot analysis also revealed that 4OHT-treated *Pdpk1^fl/fl^* Tamox*Cre* CTL had lost phosphorylation of RSK on the PDK1 target site S227 and had lost phosphorylation of Akt on the PDK1 target site T308. The loss of Akt and S6K1 catalytic activity was judged by the loss of phosphorylated Foxos and the ribosomal S6 subunit (Figure 2B).

*Pdpk1* deletion in CTL thus eliminated the activity of multiple AGC kinases. We therefore assessed the ability of IL-2 to sustain survival and proliferation of *Pdpk1*-null cells. Figure 2C shows that *Pdpk1*-null CTL failed to proliferate in response to IL-2. However, whereas IL-2 deprivation resulted in rapid cell death of CTL (Figure 1B), the loss of PDK1 did not cause cell death (Figure 2D). PDK1 is thus essential for IL-2-induced T cell proliferation but not for cell survival.

### IL-2 and PDK1 but Not Akt Sustain Glucose Uptake in CTL

**Figure 3:**
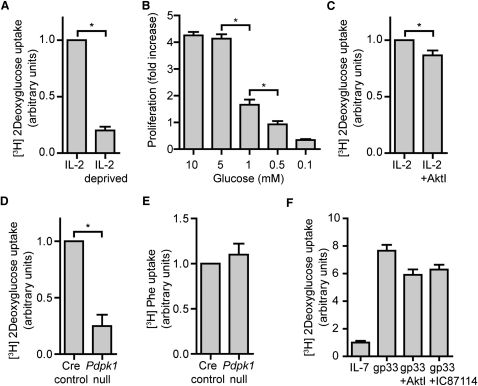
IL-2 and PDK1 but Not Akt Sustain Glucose Uptake in CTL (A) 2-deoxyglucose (2DOG) uptake by wild-type splenic T cells activated with 2C11 for 48 hr and then cultured in IL-2 for 5 days, with some cells being deprived of IL-2 for the last 24 hr of culture. (B) Wild-type splenocytes were activated with 2C11 and IL-2 for 2 days and then cultured in IL-2 in standard media for 72 hr. For the final 48 hr of culture, cells were placed in media containing indicated glucose concentrations and the fold change in cell number assessed. (C) Relative 2DOG uptake in wild-type splenic T cells activated with 2C11 for 48 hr then cultured in IL-2 for an additional 5 days, with 1 μM AktI being added for the last 48 hr of culture. (D and E) Relative 2DOG uptake (D) and phenylalanine uptake (E) by Cre control (*Pdpk1^+/+/ tm9(creEsr1)Arte^* +4OHT) and *Pdpk1*-null (*Pdpk1^fl/fl/ tm9(creEsr1)Arte^* +4OHT) CTL. (F) Naive P14 T cells were maintained in IL-7 or stimulated for 18 hr with gp33 in the presence or absence of AktI or IC87114 and then their ability to take up 2DOG was assayed. Data are representative of 3 (B, D, F), 5 (A), or 11 (C) experiments; mean ± SEM of biological replicates, except (F), representative data from 2 experiments; mean ± SD of technical replicates.

In many cells Akt controls cell proliferation because it maintains glucose metabolism. In this context, as naive CD8^+^ T cells respond to cognate antigen and differentiate to CTL, they increase glucose uptake and become highly glycolytic. Glucose uptake in CTL was not autonomous but depended on the provision of exogenous IL-2 (Figure 3A). Moreover, the importance of glucose uptake for T cell proliferation was easily demonstrated: lowering of exogenous glucose concentrations severely impaired IL-2-induced proliferative responses (Figure 3B). That glucose deprivation but not Akt inhibition prevented IL-2-induced CTL proliferation argues that Akt activity is not required for IL-2-driven glucose uptake. Figure 3C addresses this point and shows that loss of Akt activity had no impact on the ability of IL-2-cultured CTL to take up glucose. The Akt independence of IL-2-induced T cell proliferation can thus be explained by the ability of IL-2 to maintain glucose uptake via Akt-independent pathways. We then assessed whether PDK1 plays a role in IL-2-induced glucose uptake. Figure 3D shows that PDK1-null T cells had a strikingly reduced rate of glucose uptake but no defects in amino acid uptake (Figure 3E). Hence PDK1, but not Akt, is essential for IL-2 control of glucose uptake.

The Akt independence of IL-2-induced glucose uptake raised the question whether Akt activity was required for the initial increase in glucose metabolism initiated by the TCR in CD8^+^ T cells. Figure 3F shows that TCR triggering stimulated a large increased glucose uptake and this was unimpaired in cells treated with the Akt inhibitor AktI or the PI3K p110δ inhibitor IC87114. The regulation of glucose uptake via the T cell antigen receptor in CD8 T cells is thus not mediated by Akt.

### PDK1 and Akt Transcriptional Programs in CTL

If Akt is not essential for glucose uptake or IL-2-mediated T cell survival or proliferation, what does it do? This question is important because sustained IL-2 signaling is required to maintain CTL effector function both in vitro and in vivo (Kalia et al., 2010; Pipkin et al., 2010) and there is clearly the potential for Akt to mediate the effects of IL-2 on CTL function. Accordingly, we used Affymetrix microarray analysis to transcriptionally profile *Pdpk1*-null and Akt-inhibited CTL. Approximately 9400 annotated genes were expressed in CTL, and the impact of PDK1 loss or Akt inhibition was revealed by a decrease in the expression of less than 2%–3% of these transcripts and an increase in the expression of another 3%–4% (Tables S1 and S2 available online).

**Figure 4:**
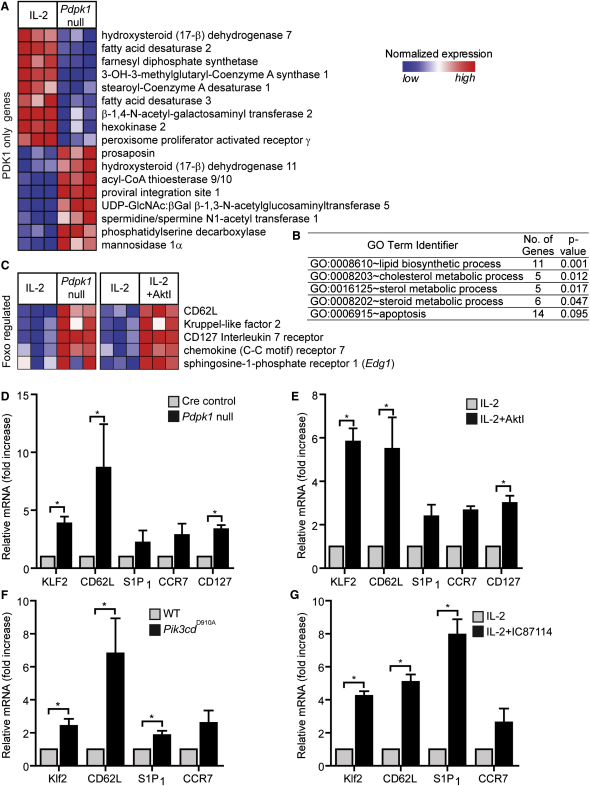
PDK1 and Akt Regulation of the CTL Transcriptional Program *Pdpk1*-null (*Pdpk1^fl/fl/ tm9(creEsr1)Arte^* +4OHT) and Cre control (*Pdpk1^+/+/ tm9(creEsr1)Arte^* +4OHT) CTL were generated and their transcriptional profiles compared via microarray. Analysis was performed with cells from three age- and sex-matched mice for each genotype. In a separate experiment, three wild-type (C57/BL6) age- and sex-matched mice were used to generate CTL by activating wild-type splenocytes with 2C11 for 48 hr with IL-2 then culturing the cells in IL-2 for a further 5 days, with 1 μM AktI being added to half of each culture for the last 48 hr. Comparison of the transcriptional profile in control versus AktI-treated cells was then performed by microarray. (A) Heat map showing the relative normalized expression of selected genes changing in expression after *Pdpk1* loss but not Akt inhibition. (B) Gene ontology analysis of genes changing in expression after *Pdpk1* loss but not Akt inhibition. (C) Heat maps showing the relative normalized expression of Foxo-regulated genes that are significantly different in expression between untreated and AktI-treated CTL and between Cre control and *Pdpk1*-null CTL. (D–G) Real-time PCR measurements of relative gene expression levels in (D) Cre control versus *Pdpk1*-null CTL, (E) untreated versus 48 hr AktI-treated wild-type CTL, (F) wild-type versus PI3K p110δ^D910A^-expressing CTL (*Pik3cd*^D910A^), and (G) untreated versus 48 hr IC87114-treated CTL. Data representative of a minimum of three experiments; mean ± SEM of biological replicates.

We first looked for non-Akt-dependent, PDK1-regulated genes to understand why PDK1 but not Akt is required for IL-2-induced T cell proliferation. Selected genes controlled only by PDK1 but not Akt are highlighted in Figure 4A (Table S3). Of particular note, PDK1 controlled expression of hexokinase 2, and hexokinases play a key role in effective uptake of exogenous glucose. We also used the NIAID DAVID website (http://david.abcc.ncifcrf.gov) to mine the gene ontology terms (GO) associated with the PDK1-controlled genes. We noted that GO terms related to lipid and cholesterol metabolism (Figure 4B) were strongly overrepresented in this list, indicating that PDK1 but not Akt controls lipid metabolism in CTL.

We next interrogated the data to characterize genes coregulated by PDK1 and Akt. One clear result was that Akt inhibition or *Pdpk1* deletion resulted in the re-expression of Foxo target genes (Figure 4C), including the IL-7 receptor α subunit (CD127), the transcription factor Klf2, and its targets L-selectin (CD62L), CC motif chemokine receptor 7 (CCR7), and sphingosine-1-phosphate receptor 1 (S1P1) (Carlson et al., 2006; Fabre et al., 2008; Kerdiles et al., 2009; Ouyang et al., 2009). Figures 4D and 4E used quantitative polymerase chain reaction (PCR) analysis to verify these changes. IL-2 activation of Akt is dependent on PI3K p110δ (Figure 1), so it would be predicted that inactivating p110δ would also restore expression of the Foxo-regulated genes in CTL. Indeed, IL-2-cultured CTL expressing catalytically inactive p110δ (*Pik3cd*^D910A^) maintained high expression of Klf2 and CD62L, CCR7, and S1P1 (Figure 4F). Moreover, treatment of CTL with the p110δ inhibitor IC87114 drove re-expression of Klf2 and its targets (Figure 4G).

### Inhibition of PI3K p110δ and Akt Reprograms CTL Trafficking

**Figure 5:**
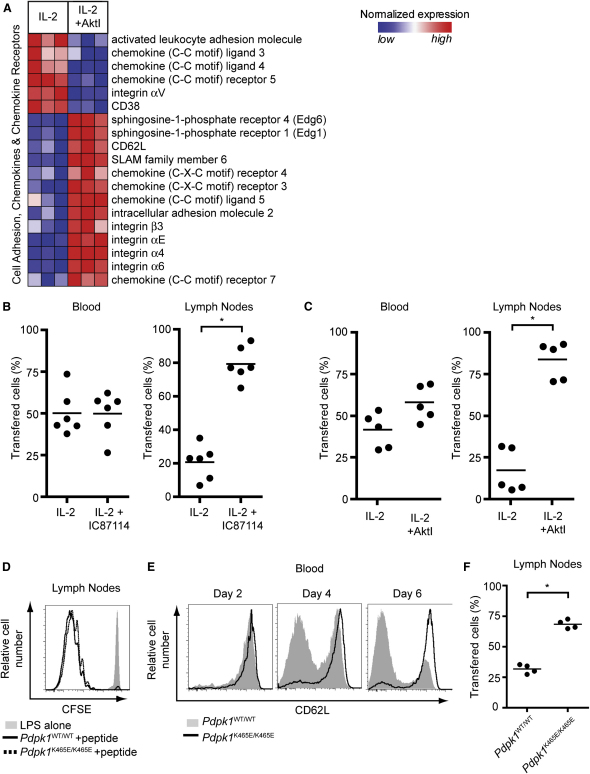
Suppression of PI3K p110δ and Akt Reprograms Lymph Node Homing of CTL (A) Heat map showing the relative normalized expression of selected genes that are significantly different in expression between untreated and AktI-treated CTL, as determined by microarray. (B) Lymph node P14 T cells were activated for 2 days with cognate peptide and then cultured for 5 days in IL-2, with IC87114 being added to half of each culture for the last 48 hr. Cells were then labeled with carboxyfluorescein succinimidyl ester (CFSE) or 5-(and-6) (((4chloromethyl)benzoyl) amino) tetramethylrhodamine (CMTMR) and mixed at a ratio of 1:1 before being injected into C57/BL6 host mice. Values indicate recovery of inhibitor-treated or untreated cells as a percentage of the total recovered transferred cells from the blood and lymph nodes 18 hr after transfer. Each dot indicates a mouse; horizontal bars indicate mean. (C) As for (B) with cells being treated with AktI rather than IC87114 for the last 48 hr of culture. (D–F) CD8^+^ T cells expressing Vα2Vβ5 TCR were purified from *Pdpk1^WT/WT^* OT1 (CD45.1^+/+^) and *Pdpk1^K465E/K465E^* OT1 (CD45.1^+^ CD45.2^+^) mice, mixed at a 1:1 ratio, and stained with CFSE. 5 × 10^6^ mixed T cells were injected into the tail vein of C57/BL6 mice (CD45.2^+/+^). 24 hr later the host mice were injected intraperitoneally with either 25 mg LPS or LPS plus 40 mg of SIINFEKL peptide. The ratio of *Pdpk1^WT/WT^* OT1 and *Pdpk1^K465E/K465E^* OT1 donor cells in lymph nodes and proliferation (CFSE dilution) was analyzed after 6 days, with CD62L expression assayed after 2, 4, and 6 days.

Deletion of *Pdpk1* or the inhibition of Akt caused CTL to re-express CD62L and CCR7 (Figures 4C–4E). CD62L controls T cell adhesion to the endothelium of high endothelial venules and CCR7 directs migration of T cells into lymphoid organs. Moreover, the loss of CD62L and CCR7 by CTL is part of the program that directs the trafficking of CTL away from lymphoid tissue. The re-expression of CD62L and CCR7 in CTL deleted of *Pdpk1* or exposed to AktI could argue that inhibition of Akt might reprogram the trafficking of CTL and restore their ability to transmigrate from the blood into secondary lymphoid tissue. In this context, strong activation of PI3K and Akt is required and sufficient to downregulate the ability of naive T cells to home to secondary lymphoid tissue in vivo (Finlay et al., 2009; Waugh et al., 2009). It is also known that p110δ activity is required for T cells to exit lymphoid tissue and home to sites of infection (Liu and Uzonna, 2010). T cells thus appear to need to activate Akt to exit lymphoid tissues. However, it is not known whether inhibition of Akt is sufficient to reprogram the ability of CTL to home to lymphoid tissue. In this context, inhibition of Akt had pleiotropic effects on the expression of multiple adhesion molecules and chemokine receptors by CTL and was not limited to controlling CD62L and CCR7 expression (Figure 5A).

To directly test the impact of Akt inhibition on CTL trafficking, in vivo adoptive transfer experiments were performed comparing the ability of wild-type CTL or CTL pretreated with either AktI or the p110δ inhibitor IC87114 to home from the blood to lymphoid tissue. Strikingly, CTL pretreated with either AktI or IC87114 showed strong preferential homing to lymph nodes and spleen compared to wild-type CTL (Figures 5B and 5C). Sustained activation of Akt is thus required to maintain the trafficking characteristics of CTL: Loss of p110δ catalytic activity or Akt inhibition reprograms the ability of CTL to traffic to lymphoid tissue.

To further explore the in vivo role of Akt in CD8 T cell trafficking responses, we compared in vivo immune responses of wild-type and *Pdpk1^K465E/K465E^* T cells. The K465E mutation creates a PDK1 molecule with a mutated PH domain that cannot bind PI(3,4,5)P_3_ and can support only a low amount of Akt activity (Waugh et al., 2009). Figure 5D shows that both wild-type and K465E T cells proliferated in vivo in response to immunization with cognate antigen and lipopolysaccharide (LPS). However, K465E T cells failed to downregulate CD62L (Figure 5E) and were retained in lymphoid tissue (Figure 5F) compared to control effector CD8^+^ T cells.

### Akt Is Necessary to Induce and Sustain Expression of CTL Effector Molecules

**Figure 6:**
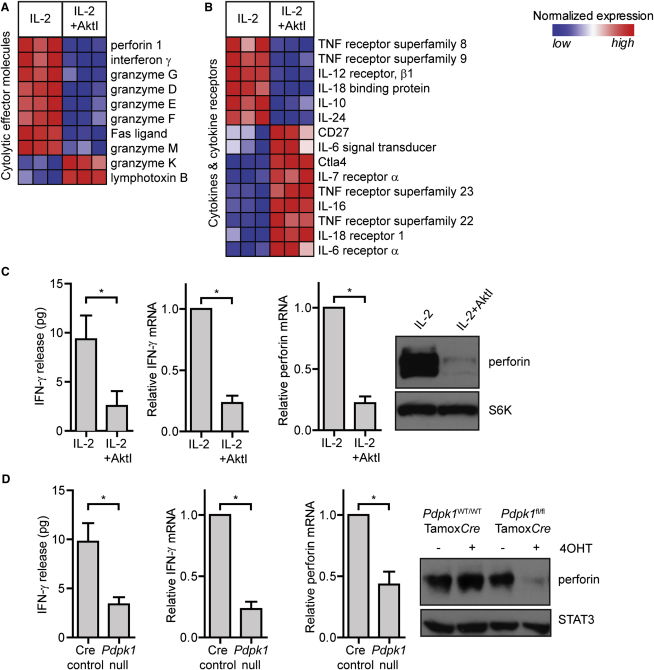
Suppression of PI3K p110δ and Akt Alters the Expression of Key CTL Effector Molecules (A and B) Heat maps showing the relative normalized expression of selected genes that are significantly different in expression between untreated and AktI-treated CTL, as determined by microarray. (C) Wild-type splenocytes were activated with 2C11 for 48 hr with IL-2 and then cultured in IL-2 for a further 5 days, with half the cells being placed in AktI for the last 48 hr of culture. Shown is IFN-γ release by 1 million cells in 6 hr, relative IFN-γ mRNA content, immunoblot analysis of total perforin protein content, and perforin mRNA content of cells cultured with and without AktI. (D) *Pdpk1*-null (*Pdpk1^fl/fl/ tm9(creEsr1)Arte^* +4OHT) and Cre control (*Pdpk1^+/+/ tm9(creEsr1)Arte^* +4OHT) CTL were generated. Shown are IFN-γ release by 1 million cells in 6 hr, relative IFN-γ mRNA content, immunoblot analysis of total perforin protein content, and perforin mRNA content of Cre control versus *Pdpk1*-null CTL. Data representative of a minimum of three experiments; mean ± SEM of biological replicates.

A key role for IL-2 in CD8^+^ T cells is to promote effector differentiation of CTL by driving expression of cytolytic effector molecules and by controlling the cytokine receptor profile of CTL (Janas et al., 2005; Pipkin et al., 2010). It was therefore striking that inhibition of Akt caused a decrease in the expression of mRNA encoding several molecules critical for CTL effector function (Figure 6A), notably multiple granzymes, Fas ligand, the cytolytic effector perforin, and interferon gamma (IFN-γ). It was equally notable that Akt inhibition had a considerable impact on the transcription of key cytokine receptors. Akt inhibition thus decreased expression of mRNA encoding IL-12 receptor β-chains while simultaneously increasing expression of mRNA encoding IL-6 receptors and CD27 (TNFRSF7), the CD70 coreceptor (Figure 6B). The role of Akt in perforin and IFN-γ expression was verified by real-time PCR analysis, ELISA, and immunoblot analysis (Figure 6C). Akt inhibition thus caused loss of IFN-γ mRNA expression and prevented IFN-γ protein production. Similar results were obtained after the deletion of *Pdpk1* from CTL (Figure 6D), providing genetic evidence that PDK1-Akt signaling sustains perforin and IFN-γ protein expression in CTL.

**Figure 7:**
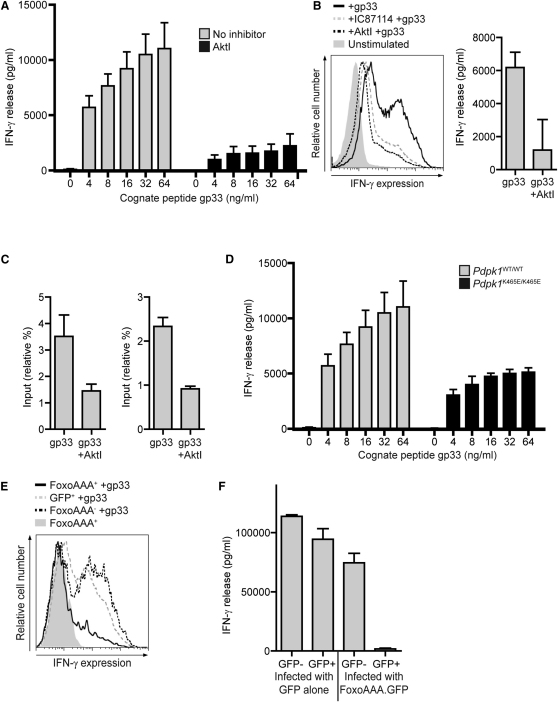
PDK1 Controls Antigen Receptor-Driven IFN-γ Expression via a Foxo-Mediated Mechanism (A) Naive P14 lymph node T cells were stimulated for 4 hr with LCMV peptide gp33 in the presence or absence of AktI and the IFN-γ produced assayed by ELISA. (B) P14 T cells were activated for 48 hr with gp33 and then cultured for 3 days in IL-2. Left: Cells were then stimulated for 4 hr with 100 ng/ml gp33 in the presence of AktI or IC87114 and IFN-γ production assayed by flow cytometry. Right: Alternatively, cells were stimulated for 4 hr with 125 ng/ml gp33 in the presence of AktI and IFN-γ production assayed by ELISA. (C) P14 T cells were activated for 48 hr with gp33 and then cultured for 3 days in IL-2. Cells were stimulated for 3 hr with 62.5 ng/ml gp33 in the presence or absence of AktI. ChIP was performed with anti-PolII, and the changes in PolII binding to (left) the IFN-γ-proximal promoter region and (right) the IFN-γ fourth exon were quantified by real-time PCR. Data are normalized to input DNA amounts and plotted as fold over the values for PolII binding to the HPRT-proximal promoter. (D) P14 TCR transgenic T cells from lymph nodes of *Pdpk1^WT/WT^* and *Pdpk1^K465E/K465E^* P14 transgenic mice were activated for 48 hr with gp33 and then cultured for 3 days in IL-2. Cells were then stimulated for 4 hr with gp33 and IFN-γ produced assayed by ELISA. (E and F) P14 T cells were activated for 24 hr with gp33 and then spinfected with retrovirus encoding either GFP or GFP-tagged Foxo3a T32A S252A S314A (FoxAAA). Three days after infection, cells were (E) stimulated for 4 hr with 100 ng/ml gp33 in the presence or absence of AktI or IC87114 and then stained for intracellular IFN-γ or (F) sorted into GFP^+/−^ populations, cultured for a further 24 hr, and then stimulated for 4 hr with 100 ng/ml gp33 and IFN-γ production assayed by ELISA. Data are representative of a minimum of two (E, F) or three (A–C, D) experiments; mean ± SEM of biological replicates.

Akt is also rapidly activated in CD8^+^ T cells in response to triggering of the TCR. Akt is not required for TCR-induced metabolic programs (Figure 3F), but is it necessary for the TCR to initiate expression of CTL effector molecules? In these experiments we focused on the induction of IFN-γ because this cytokine can be induced rapidly in response to triggering of the TCR in both naive and effector CD8^+^ T cells. Moreover, it has been shown that p110δ is required for TCR-induced IFN-γ production during immune activation but the role of Akt has not been addressed (Liu and Uzonna, 2010; Soond et al., 2010). We found that inhibition of Akt suppressed TCR induction of IFN-γ mRNA and protein in both naive CD8^+^ T cells and effector CTL (Figures 7A and 7B). This reflects that Akt activity is important for IFN-γ gene transcription. Figure 7C thus shows that the loss of Akt activity reduced the amount of RNA polymerase II (Pol II) recruitment to the IFN-γ transcription start site and distal exons.

Why is Akt needed for IFN-γ production? We considered the possibility that Akt controlled the activity of mTOR (mammalian target of rapamycin), which is known to control the expression of T effector molecules (Rao et al., 2010). However, although *Pdpk1* deletion prevented phosphorylation of the mTOR target S6 (Figure 2B), inhibition of Akt only weakly suppressed the phosphorylation of S6 and S6K1 (Figure S1). Akt activity is thus dispensable for mTOR activation in CTL. Accordingly, an inability to activate mTOR does not explain why Akt is required for IFN-γ production. However, an important insight as to a possible mechanism for Akt control of IFN-γ production came from experiments with *Pdpk1^K465E/K465E^* T cells that support only a low level of Akt activity. These T cells have a selective defect in the phosphorylation and inactivation of Foxo family transcription factors and fail to downregulate expression of Foxo gene targets (Waugh et al., 2009). Foxos can both transactivate and repress gene expression (Fabre et al., 2008), and in this context, TCR-induced production of IFN-γ is severely attenuated in *Pdpk1^K465E/K465E^* T cells (Figure 7D). This is consistent with a model whereby the defects in IFN-γ production caused by inhibition of Akt activity in CTL might be caused by relocation of Foxo transcription factors in the nuclei of T cells that then repress the IFN-γ gene. To assess whether the restoration of Foxos to the nuclei of TCR-activated T cells would impact on IFN-γ gene expression, we examined IFN-γ production in T cells expressing a GFP-tagged Foxo3a mutant with alanine substitutions at its Akt substrate sequences, T32, S252, and S314 (FoxoAAA). This phospho mutant could restore Foxo transcriptional function in cells expressing active Akt. Figures 7E and 7F show that activated T cells expressing the FoxoAAA mutant could not produce IFN-γ in response to peptide triggering of TCR complexes. Akt-regulated nuclear export of Foxo transcription factors is thus required for IFN-γ production.

## Discussion

The objective of the present study was to gain a more in-depth understanding of the function of PDK1 and Akt in the context of TCR and IL-2 signal transduction in CD8^+^ T cells. The accepted view of Akt is that it controls T cell metabolism. However, the data herein showed that Akt is not essential for CD8^+^ T cell metabolism, survival, or proliferation. Nevertheless, Akt does have a critical role in CTL function. Akt permits TCR and IL-2 signaling to maintain the expression of cytolytic effector molecules and to determine the repertoire of cytokine receptors, adhesion molecules, chemokine receptors, and effector molecules that distinguish effector and memory CTL populations. The present results thus force a shift in the accepted paradigm that Akt functions as an obligate controller of T cell metabolism. Rather, Akt simultaneously induces and represses expression of key genes to create an effector CTL, with the Foxo transcription factors being at the center of this process.

The TCR and IL-2 control the metabolic programs of CTL by driving glucose uptake, amino acid uptake, and protein synthesis. The current results show that this metabolic role is not mediated by Akt but is controlled by PDK1. This kinase phosphorylates and activates many AGC family serine kinases including Akt, PKCs, S6Ks, RSK, and the SGKs (Bayascas, 2008; Williams et al., 2000). These all share similar substrate specificities and there are numerous examples of redundant functions between different family members (Juntilla et al., 2007; Kelly et al., 2007). Importantly, the ability of PDK1 to phosphorylate the RSKs and the SGKs is independent of PI3K signaling (Bayascas et al., 2008; Waugh et al., 2009). Accordingly, PDK1 loss is more global than inhibition of PI(3,4,5)P_3_ production for terminating AGC kinase activity. The fact that glucose uptake and proliferation of CTL is PDK1 dependent rather than Akt dependent thus reveals that there is redundancy between AGC family kinases in CD8^+^ T cells. These data also indicate that PI3K and Akt do not have obligatory functions as metabolic switches in lymphocytes because other molecules can assume this role. These include other PDK1-controlled molecules, but it is also noteworthy that deletion of the serine-threonine master kinase LKB1 causes death of activated T cells (Tamás et al., 2010). There is also evidence that PIM kinases are important regulators of T cell survival (Fox et al., 2005). The control of T cell metabolism may thus be determined by multiple kinases.

Akt may not control CTL metabolism but the data herein shown that Akt does control a fundamental part of the CTL transcriptional program. Here it is relevant that cytotoxic T cells have two fates: they continue to become terminally differentiated “exhausted” effector T cells destined to die during the contraction phase of the immune response or a few may divert to become memory T cells (Ahmed et al., 2009). TCR triggering initiates CTL differentiation and sustained IL-2 signaling promotes effector CTL differentiation. The present data now show that the ability of these receptors to sustain Akt activity is needed to maintain the transcriptional program of CTL. In particular, Akt signaling maintained CTL effector function and controlled expression of multiple biomarkers that distinguish central memory T cells from effector T cells. The loss of Akt activity thus reprogrammed CTL to assume a “memory-naive” phenotype by synchronizing a decrease in the expression of cytolytic effector molecules such as perforin and IFN-γ while causing an increase in expression of genes encoding the cytokine receptors for IL-6 and IL-7 along with tumor necrosis factor receptor family member CD27. These latter molecules all play key roles in the homeostasis and survival of long-lived memory CD8^+^ T cells (Castellino and Germain, 2007; Hendriks et al., 2003; Kaech et al., 2003). Akt also controlled the repertoire of chemokine and adhesion molecules expressed by T cells that define whether these cells can traffic to secondary lymphoid tissue and progress to memory cells. Importantly, Akt inhibition allowed CTL to reacquire the ability to home to secondary lymphoid tissue. Collectively, these results reveal that sustained Akt signaling is required to maintain CTL effector function; the loss or reduction of Akt signaling does not impact T cell survival or proliferation but causes differentiated CTL to transcriptionally reprogram from an effector to a memory phenotype. Hence the simple view of Akt as a metabolic regulator is likely to be incorrect; rather, in CTL the role of Akt is to control the transcriptional programs that direct effector versus memory T cell fate. In particular, the data regarding Akt and T cell migration afford an explanation for the defects in the ability of antigen-primed PI3K p110δ-deficient T cells in vivo to migrate to antigenic sites (Jarmin et al., 2008; Liu and Uzonna, 2010). The data also argue that the in vivo role of IL-2 to sustain effector CTL function may rely on Akt-dependent expression of differentiation and trafficking molecules.

Finally, an emerging concept in T cell biology is that limiting T cell metabolism may accelerate the conversion of effector CTL into a memory subset (Araki et al., 2009; Pearce et al., 2009; Prlic and Bevan, 2009). This idea was based on studies linking mammalian target of rapamycin (mTOR) and adenosine monophosphate-activated protein kinase (AMPK) to the production of memory T cells during an immune response (Araki et al., 2009; Pearce et al., 2009; Rao et al., 2010). AMPK and mTOR are known to regulate cell metabolism but it was not directly proven that the role of either enzyme was caused by effects on T cell metabolism. The role of Akt in CTL suggests an alternative possibility: enzymes that originally evolved to control cell metabolism have evolved the ability to control other aspects of T cell function and can do so independently of effects on T cell metabolism.

## Experimental Procedures

### Mice

C57BL/6GT(ROSA)26^tm9(creEsr1)Arte^ (Tamox*Cre*) mice were obtained from Taconic Artemis Pharmaceuticals. Mice carrying floxed *Pdpk1* alleles *Pdpk1^flΔneo^* (PDK1^fl^); OT1 and P14 TCR transgenic mice; P14 TCR transgenic mice carrying a knockin mutation for a substitution of lysine for glutamic acid at residue 465 in the PH domain of PDK1 (*Pdpk1^K465E^*) and *Pik3cd*^D910A^ mice containing a knockin mutation of PI3K wherein wild-type alleles of the p110δ catalytic subunit of PI3K were substituted with a point mutation (D910A), which is a catalytically inactive form of p110δ (p110δ^D910A^), have been described (Kelly et al., 2007; Kurts et al., 1996; Pircher et al., 1989; Waugh et al., 2009). Mice were produced in the Biological Resource Unit at the University of Dundee in compliance with UK Home Office Animals (Scientific Procedures) Act 1986 guidelines.

### Cell Culture

CD8^+^ T cells were isolated from spleens and/or lymph nodes with an AutoMACs magnetic cell sorter (Miltenyi Biotec). Lymphocytes were suspended in RPMI 1640 plus 10% heat-inactivated fetal calf serum (FCS), penicillin, streptomycin (GIBCO), and 50 μM β-mercaptoethanol (β-ME) (Sigma) and activated for 48 hr in either 0.5 μg/ml CD3 antibody (2C11) or 100 ng/ml LCMV TCR-specific peptide gp33-41, KAVYNFATM. Cells were then washed free of activating agent and thereafter maintained in 20 ng/ml IL-2 at a density of approximately 0.3 × 10^6^/ml at 37°C, 5% CO_2_. 4-hydroxytamoxifen (4OHT, Sigma) was used at 0.6 μM. AktI-1/2 (AktI) (Calbiochem) was used at 1 μM, the p110δ inhibitor IC87114 (made in-house) and LY294002 (Promega) were used at 10 μM. Where indicated cells were cultured in media of defined glucose concentrations with glucose-free RPMI containing L-glutamine (GIBCO) with 10% dialyzed FCS (GIBCO), penicillin, streptomycin, 50 μM β-ME (glucose-free media) plus D(+)glucose (Sigma). *Pdpk1*-deleted CTL were prepared by activating *Pdpk1*^fl/fl^ Tamox*Cre* splenocytes with 2C11 for 2 days and then culturing the cells in IL-2 for 5 days, with 0.6 μM 4OHT being added for the final 72 hr of culture.

### Flow Cytometry

Cells were labeled in RPMI plus 0.5% FCS with Phycoerythrin-conjugated anti-CD62L, Alexa750-conjugated anti-CD4, PerCP.Cy5.5-conjugated anti-TCR Vα2, allophycocyanin-conjugated anti-CD45.2, Horizon V450-conjugated anti-CD45.1, and phycoerythrin.Cy7-conjugated anti-CD8. For FoxoAAA, IFN-γ double-staining cells were stimulated in the presence of 3 μg/ml Golgiplug (BD), fixed and permeabilized as per manufacturer's instructions (eBioscience 00-8222-49, 00-8333-56), and then stained with Alexa488-conjugated anti-GFP (Invitrogen) plus allophycocyanin-conjugated anti-IFN-γ (BD Biosciences). Data were acquired on a FACS Calibur (BD Biosciences) and analyzed with FlowJo software (Treestar). Viable cells were gated according to their forward scatter (FSC) and side scatter (SSC) profiles. GFP^+/−^ cell isolation was performed with a Vantage cell sorter (BD Biosciences).

### Immunoblot Analysis

Cells were lysed on ice in HEPES lysis buffer: 100 mM HEPES (pH 7.4), 150 mM NaCl, 20 mM NaF, 20 mM iodoacetamide, 2 mM EDTA, 1% NP-40, 1 mM phenylmethylsulfonyl fluoride, 1 mM sodium orthovanadate, 40 mM β-glycerophosphate (Sigma), and protease inhibitors (Roche). Lysates were centrifuged (4°C, 1600 × g, 15 min) and then samples separated via sodium dodecyl sulfate 4%–12% polyacrylamide gel electrophoresis and transferred to nitrocellulose membranes. Blots were probed for pT24 Foxo1 + p32Foxo3a; pT308 and total Akt; pT235 + pT236 and total small ribosomal subunit S6; pS389, pS421 + pS424, and total S6K; PKCθ (all Cell Signaling Technologies); pS227 p90RSK (Santa-Cruz Biotechnology); total Foxo3a (made in house); PDK1 (Upstate); or perforin (gift of G. Griffith).

### Glucose and Phenylalanine Uptake

1 × 10^6^ cells were suspended in 500 μl glucose-free media for 10 min. 500 μl glucose-free media containing 1 μCi/ml 2-Deoxy-D-[1-^3^H]glucose ([^3^H] 2DOG) (GE healthcare) was then added and the cells incubated for a further 10 min. Cells were pelleted, washed in PBS, and then lysed in water. Lysate ^3^H content was then measured via liquid scintillation counting. For phenylalanine uptake, the assay was performed essentially as for glucose uptake, except that assays were performed in HBSS, 50 μM β-ME, 1 × MEM vitamins (GIBCO), with 10% dialyzed FCS. [^3^H]Phe (GE Healthcare) was added and cells incubated for 1 hr prior to pelleting.

### Real-Time PCR

RNA was extracted from cells with the RNeasy RNA purification minikit (QIAGEN). Reverse-transcription PCR was performed with qScript cDNA synthesis kit (Quanta). Real-time PCR was performed with iQ SYBR Green detection chemistry (BioRad) on an iCycler (BioRad). Relative mRNA levels of genes of interest were normalized to hypoxanthine guanine phosphoribosyltransferase (HPRT). Primers are detailed in the Supplemental Experimental Procedures.

### ChIP

Real-time PCR-based chromatin immunoprecipitation (ChIP) analysis to measure RNA Pol II binding to the IFN-γ locus was performed described (Cruz-Guilloty et al., 2009) with minor modifications. Chromatin was immunoprecipitated with anti-Pol II (Santa-Cruz Biotechnology) or normal rabbit IgG (Cell Signaling) from 5 × 10^6^ cells in presence of 0.2 mg/ml BSA. ChIP Grade Protein G Magnetic Beads (Cell Signaling) were used to collect the immunocomplexes. Chromatin was purified via a NucleoSpin Extract II kit (Macherey-Nagel) and resuspended in TE buffer. Real-time PCR was performed in a Biorad iQ5 with Perfecta SYBR green FastMix for iQ (Quanta BioSciences). Primers are detailed in the Supplemental Experimental Procedures.

### Retroviral Transduction

The production of FoxoAAA-GFP (Foxo3a T32A S252A S314A) and control retrovirus has been described previously (Waugh et al., 2009). The retroviral infection protocol was performed as described previously (Waugh et al., 2009).

### IFN-γ Quantification

IFN-γ was assayed in culture supernatants with the femto-HS high-sensitivity IFN-γ ELISA kit (eBiosciences).

### Microarray Analysis

Microarray analysis was carried out by the Finnish DNA Microarray Centre at the Centre for Biotechnology, Turku, Finland via 430_2.0 mouse expression arrays (Affymetrix) and the manufacturer's recommended protocol. Statistically significant differences in gene expression were identified with Multiple Experiment Viewer v4.3 (Saeed et al., 2003). Overrepresented gene ontology terms were identified with the NIAID DAVID website (http://www.david.abcc.ncifcrf.gov). Full details are given in Supplemental Experimental Procedures.

### Statistical Analyses

With the exception of the microarray data, statistical analyses were performed with Prism 4.00 for Macintosh (GraphPad). A nonparametric Mann Whitney test was used where the number of experiments performed was not sufficient to prove normal distribution. Statistically significant results (p < 0.05) are indicated by an asterisk.

## Accession Numbers

The microarray data are available in the Gene Expression Omnibus (GEO) database (http://www.ncbi.nlm.nih.gov/gds) under the accession number GSE26290.

## Supplemental Information

Document S1. Supplemental Experimental Procedures and One Figure

Table S1. Genes Changing Significantly in Expression in CTL after Pdpk1 Deletion

Table S2. Genes Changing Significantly in Expression in CTL after Akt Inhibition

Table S3. Genes Changing Significantly in Expression in CTL after Pdpk1 Deletion but Not After Akt Inhibition
