# Supplementary material for: Protein Kinase B Controls Transcriptional Programs that Direct Cytotoxic T Cell Fate but Is Dispensable for T Cell Metabolism
Source: Immunity. 2011 Feb 25;34(2):224–36. doi: 10.1016/j.immuni.2011.01.012 (PMC3052433; doi:10.1016/j.immuni.2011.01.012)
Supplement: Document S1. Supplemental Experimental Procedures and One Figure [file mmc1.pdf]

Immunity, Volume *34*

Supplemental Information

Protein Kinase B Controls Transcriptional Programs that Direct Cytotoxic T Cell Fate but Is Dispensable for T Cell Metabolism

Andrew N. Macintyre, David Finlay, Gavin Preston, Linda V. Sinclair, Caryll M. Waugh, Peter Tamas, Carmen Feijoo, Klaus Okkenhaug, and Doreen A. Cantrell

Supplemental Experimental Procedures

rtPCR primers:

CD127: 5'CAATGCCCCTCCACTCCTTTC3' and 5'GGTGACATACGCTTCTTCTTGATT3', CCR7: 5'CAGGCTTCCTGTGTGATTTCTACA3' and 5'ACCACCAGCACGTTTTTCCT3', CD62L: 5'ACGGGCCCCAGTGTCAGTATGTG3' and 5'TGAGAAATGCCAGCCCCGAGAA3', perforin: 5'CGTCTTGGTGGGACTTCAG3' and 5'GCATTCTGACCGAGGGCAG3', IFNγ: 5'TTACTGCCACGGCACAGTC3' and 5'AGATAATCTGGCTCTGCAGG3', S1P1: 5'GTGTAGACCCAGAGTCCTGCG3' and 5'AGCTTTTCCTTGGCTGGAGAG3', Klf2: 5'TGTGAGAAATGCCTTTGAGTTTACTG3' and 5'CCCTTATAGAAATACAATCGGTCATAGTC3', HPRT: 5'TGATCAGTCAACGGGGGACA3' and 5'TTCGAGAGGTCCTTTTCACCA3'

ChIP Primers

Primers: IFNγ proximal promoter: 5'ACTCTAACATGCCACAAAACCATAG3' and 5'CTTCCAGTTTTATACCTGATCGAAG3'; IFNγ 4th exon: 5'CAAGCATTCAATGAGCTCATC3' and 5'CTTATTGGGACAATCTCTTCC3;

HPRT proximal promoter: 5'GCGTTTCTGAGCCATTGCTG3' and 5'GCTCCGGAAAGCAGTGAG-3' were used as the normalizing standard. Primers directed to the β-Globin locus were used as negative control: 5'TCTGATGGGCACCTCCTGGGT3' and 5'CCATCAACATAACTGTAGAGC3'.

Microarray Analysis

RNA was extracted as described. RNA was processed and hybridized to GeneChip mouse genome 430_2.0 array (Affymetrix) at 45°C overnight using the manufacturer's protocol. Arrays were washed and stained using a GeneChip fluidics station 450 (Affymetrix) and then scanned using a GeneChip scanner 3000 7G (Affymetrix). Microarray data are deposited in the GEO database, accession number GSE26290. Data was normalized using the Affymetrix Expression Console v1.1 (Affymetrix) implementation of the robust multichip averaging algorithm and then analyzed further in Multiple Experiment Viewer v4.3 (Saeed et al., 2003). Statistically significant differences in gene expression were identified using the SAM algorithm (Tusher et al., 2001), identifying genes changing ≥1.5 fold with a 90th percentile false discovery rate set to 5%. Gene ontology (GO) terms over-represented in the list of statistically significant gene changes were identified using the NIAID DAVID website (david.abcc.ncifcrf.gov). Over-represented 5th level GO terms describing 'biological process' annotation were identified using an EASE score cut-off $p<0.1$. Terms represented by <5 genes in the changed gene list were excluded from the analysis. Heatmaps of relative normalized expression data were generated using the GenePattern Software suite (genepattern.org).

Flow cytometry analysis of S6 phosphorylation

Intracellular phosphorylation of small ribosomal subunit S6 on T235/236 was performed as previously described (Hinton et al., 2006).

References

Hinton, H.J., Clarke, R.G., and Cantrell, D.A. (2006). Antigen receptor regulation of phosphoinositide-dependent kinase 1 pathways during thymocyte development. FEBS Lett *580*, 5845-5850.

Saeed, A.I., Sharov, V., White, J., Li, J., Liang, W., Bhagabati, N., Braisted, J., Klapa, M., Currier, T., Thiagarajan, M., *et al.* (2003). TM4: a free, open-source system for microarray data management and analysis. Biotechniques *34*, 374-378.

Tusher, V.G., Tibshirani, R., and Chu, G. (2001). Significance analysis of microarrays applied to the ionizing radiation response. Proc Natl Acad Sci U S A *98*, 5116-5121.

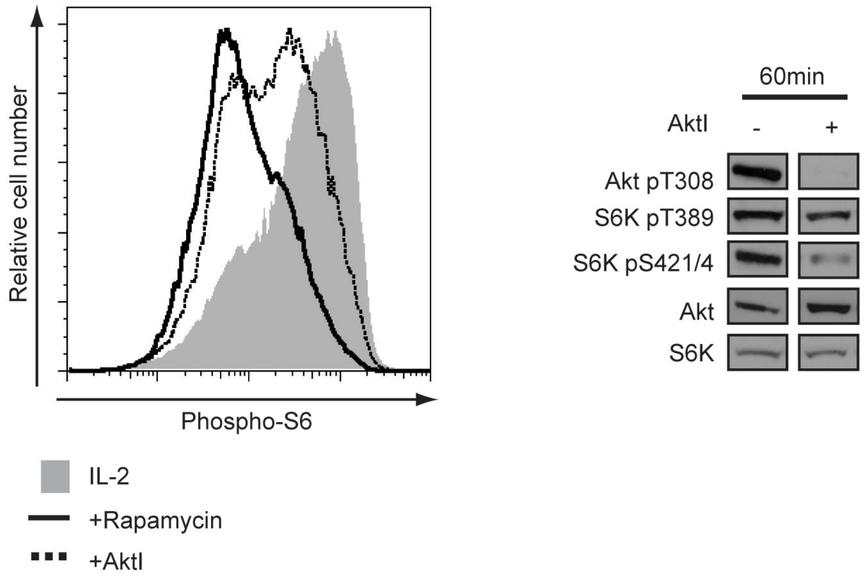

Supplementary Fig S1. Inhibition of Akt only weakly suppresses the phosphorylation of S6 and S6K1. Splenic T-cells from wild-type mice (C57/BL6) were stimulated with 0.5μg/ml CD3 antibody plus 20ng/ml IL-2 for 48 hr. Cells were then cultured for 4 days with 20ng/ml IL-2. Cells were treated with 20nM rapamycin or 1mM AktI for 60min and then either fixed and permeablised for intracellular phospho-S6 analysis by flow cytometry or lysed and analysed by western blot techniques.
